# Construction and validation of a immune-related prognostic gene DHRS1 in hepatocellular carcinoma based on bioinformatic analysis

**DOI:** 10.1097/MD.0000000000035268

**Published:** 2023-10-20

**Authors:** Sa Xu, Wei Wang, Tao Meng, Fuyan Wang, Guoxing Wang, Fan Huang, Guobin Wang, Xiaojun Yu, Ruolin Wu, Liujin Hou, Zhenghui Ye, Xinghua Zhang, Hongchuan Zhao, Yuxian Shen

**Affiliations:** a School of Basic Medical Sciences, Anhui Medical University, Hefei, China; b Department of General Surgery, First Affiliated Hospital of Anhui Medical University, Hefei, China; c Organ Transplant Center of The First Affiliated Hospital of Anhui Medical University, Hefei, China; d Department of General Surgery, Third Affiliated Hospital of Anhui Medical University, Hefei, China; e Anhui BioX-Vision Biological Technology Co., Ltd, Hefei, China.

**Keywords:** DHRS1, hepatocellular carcinoma, immunity, prognosis

## Abstract

A member of the short-chain dehydrogenase/reductase superfamily (DHRS1, SDR19C1) is a member of the short-chain dehydrogenase/reductase superfamily and a potential predictor of hepatocellular carcinoma (HCC). However, the role of DHRS1 in HCC immunity remains unclear. We systematically analyzed the association between DHRS1 and HCC immunity with transcriptional and clinical data from the Tumor Immune Estimation Resource, an integrated repository portal for tumor immune system interactions, and cBioPortal databases. Six DHRS1-associated immunomodulators strongly correlated with survival and were uncovered by exploiting univariate and multivariate Cox analyses. We created a risk score for each patient by adding the points from each immunomodulator and then classified them into high and low risk categories. Survival analysis were used to compare the overall survival between the 2 groups, and the receiver operating characteristic curve was applied to assess the accuracy of the risk score. Data from our center were adopted as the external validation set, the risk score was calculated using the risk coefficient of the 6 genes in the training cohort, and survival analysis were executed to verify the experimental group results. A nomogram was ultimately constructed with the R package. Our data revealed a correlation between the levels of immune cell infiltration and either the DHRS1 gene copy numbers or mRNA levels in HCC. Second, we generated a signature based on the 6 DHRS1-related immunomodulators (KDR, TNFRSF4, CD276, TNFSF4, SLAMF6, and SIGLEC9). We postulate that the generated risk scores would serve as an independent indicator of HCC prognosis, with an area under the receiver operating characteristic curve for the risk score of 0.743. We further established external validation sets to reconfirm the predictive validity of the risk score. Finally, a prognostic nomogram and calibration curve were created. The DHRS1 gene may exert an impact on HCC immunity. We posit that the nominated immune signature based on DHRS1-associated immunomodulators could constitute a promising prognostic biomarker in HCC.

## 1. Introduction

According to the most recent official cancer data, there were approximately 906,000 new primary liver cancers and 830,000 deaths in 2020, with hepatocellular carcinoma (HCC) accounting for approximately 75% to 85% of the total. The prevalence of HCC was greatest in areas of East Asia, and men died at substantially greater rates than women.^[1]^ Because patients are asymptomatic in the early stages of hepatocellular carcinoma, HCC proceeds to an advanced stage or even metastasizes by the time it is detected, and when the greatest opportunity for localized therapy has passed (e.g., after hepatectomy, TACE, and radiofrequency ablation).^[2]^ However, patients with HCC exhibit an appalling prognosis, with a high rate of recurrence and a poor response to chemoradiotherapy. As a result, <10% of HCC patients live for 5 years.^[3]^ The hepatic microenvironment affects immunosurveillance, which can either hamper or foster HCC development, and the immunological state of the tumor microenvironment (TME) may thus affect the prognosis of HCC. For example, Treg infiltration in HCC abrogates the immune system of the tumor, such that the presence of abundant Treg infiltration constitutes an untoward prognosis for the tumor.^[4]^ As immune checkpoints have been associated with tumor cell growth and escape from the immune system,^[5]^ immune checkpoint inhibitors such as PD-1/PDL-1 have been authorized for cancer treatment to increase immune control over malignant cells. NCCN guidelines suggest the adoption of immune checkpoint inhibitors for advanced HCC because of their outstanding effectiveness.^[6]^ Current clinical categorization, such as the tumor, node, and metastasis (TNM) staging system, however, provides no tumor immune information; therefore, it is not possible to predict the response to immune therapy.^[7]^ Consequently, selecting a biomarker related to HCC immunity as an indicator of prognostic assessment is critical.

The short-chain dehydrogenase/reductase (SDR) family member 1 (a member of the short-chain dehydrogenase/reductase superfamily [DHRS1], also known as SDR19C1) has been localized to the nucleus, mitochondria, and endoplasmic reticulum of HeLa cells. The human gene DHRS1 was found on chromosome 14q12, with its highest level of protein expression in the liver.^[8]^ In HCC, DHRS1 expression falls, and patients with reduced DHRS1 expression show a poorer prognosis and survival. Although it has not yet been demonstrated experimentally, some authors suggest that DHRS1 may constitute a unique and independent predictive biomarker and a potential therapeutic target for HCC.^[9]^ Prognostic prediction models based on DHRS1-related immune genes have been established, which have proven to be both effective and reliable, suggesting a role for DHRS1 in the infiltration of immune cells in HCC. However, to our knowledge, no relevant studies on this topic have been published.

In the present investigation, we performed a search of a publicly accessible database to analyze the relationship between DHRS1 and immunity in HCC. The cancer genome atlas (TCGA)-LIHC database was used to screen for immunomodulators associated with DHRS1, and we subsequently generated a prognostic model to predict the prognosis of HCC patients. We then conducted an external validation using 62 cases of HCC at our center.

## 2. Materials and methods

### 2.1. Patients and sample tissues

Between January 2010 and May 2015, we collected fresh tissue specimens as well as clinical and prognostic data from 62 patients who were diagnosed with HCC and who underwent radical resection for HCC at Anhui Medical University’s First Affiliated Hospital. The Ethics Committee of Anhui Medical University’s First Affiliated Hospital approved our protocol, and the IRB number was 20131359. We received informed consent from participants in our study, and we adopted clinical HCC specimens in compliance with the Declaration of Helsinki.

### 2.2. Data sources and processing

Gene expression and clinical data of patients with HCC were gathered from the TCGA-LIHC database (http://portal.gdc.cancer.gov/repository).^[10]^ The limma package in R was applied to conduct analyses on the RNA expression data.^[11]^ The Kaplan–Meier plotter method was employed to generate survival curves and confirm survival differences across groups.

### 2.3. Tumor immune cell infiltration

The tumor immune estimation resource (TIMER) (http://timer.cistrome.org/) is an interactive web tool that allows for the comprehensive and flexible analysis and visualization of tumor-infiltrating immune cells. TIMER deduces the abundance of tumor-infiltrating immune cells (B cells, CD4 + T cells, CD8 + T cells, macrophages, neutrophils, and dendritic cells) from gene expression profiles of different cancer types in the TCGA.^[12]^ In this study, the correlations between DHRS1 expression levels/copy number alterations and 6 types of immune cells were estimated using the TIMER “immune association” modules.

### 2.4. Gene set enrichment analysis

To classify patients into high and low-expression groups, we measured the median DHRS1 expression using LIHC RNA-seq. The gene set enrichment analysis (GSEA) software’s reference molecular signature database (MSigDB) (version 4.1.0) was then applied to investigate the potential regulatory pathways in which DHRS1-related genes participated.

### 2.5. Immunomodulators and related genes

Tumor immune system interactions (TISIDB) is a website in which data from a variety of sources are collected and integrated, including PubMed, high-throughput screening, TCGA, and other public databases such as Uniprot, GO, and DrugBank. We downloaded information on tumor-infiltrating lymphocytes (TILs) and immunomodulators related to DHRS1 from the TISIDB database for tumor immune interactions and sought correlations between them. (http://cis.hku.hk/TISIDB/).^[13]^ Then, DHRS1-associated immunomodulators were used to construct a protein-protein interaction network using the search tool for the retrieval of interaction gene/proteins database (https://string-db.org/). Afterward, DHRS1-associated immunomodulators were subjected to GO and KEGG analysis using the consensus online tool with FDR < 0.05 (http://cpdb.molgen.mpg.de/).

### 2.6. Construction of the prognostic signature and nomogram

The association of DHRS1-associated immunomodulators and prognosis were analyzed by stepwise Cox regression analysis using the R package “survmine”. Using DHRS1-associated immunomodulators, we constructed a prognostic multiple immune gene signature model,^[14]^ and our risk score was created by screening the associated immune genes using multivariate Cox regression to establish a prognostic index: risk score = β1 × 1 + β2 × 2 +. + βixi.^[15]^ We accounted for each gene’s expression level (xi) in this formula and designated Cox regression coefficients for each gene by βi. A stepwise Cox analysis was used to identify independent prognostic factors. The Kaplan–Meier survival curves were ultimately generated to evaluate the relationship between the risk score and overall survival (OS),^[16]^ and time-dependent receiver operating characteristic (ROC) curves^[17]^ were used to determine the prognostic accuracy of the risk scores.

### 2.7. RNA extraction and qPCR

Total RNA was isolated from HCC tissues using TRIzol reagent as directed by the manufacturer, and then reverse-transcribed into cDNA using a Prime ScriptTM RT Reagent Kit (AG, Changsha, China). Using GAPDH as an internal reference, the Ct method was used to determine the relative changes in mRNA.^[18]^ (see Table S1, Supplemental Digital Content, http://links.lww.com/MD/K398, which demonstrates the primer sequences).

### 2.8. Construction of nomogram T

A nomogram was specifically developed to visualize our statistical prediction models, and we routinely used it to present results from logistic regression or COX regression analyses. The nomogram was created for R software via the rms package. The bootstrap method was utilized in conjunction with a calibration curve (1000 replicates) to visualize the difference between predicted and actual probabilities. The forecasting precision of a nomogram was measured using the concordance index. Based on the results of the regression, we produced multiple lines at a specific scale, and the graphs were used to conveniently deduce the risk or probability of an individual’s survival. Each of the characteristics was rated separately, including age, sex, TNM staging, and risk score. Then these variables were combined to generate a total score and to allow estimation of the calibration curve for 1-, 3-, and 5-year OS.^[19]^

### 2.9. Statistical analysis

All statistical analyses were performed by R version 4.0.3 and the GSEA analysis software (GSEA Desktop Application v4.1, Broad Institute, Inc, Cambridge, MA). Outcomes were implemented using SPSS (version 22.0), GraphPad Prism (version 8.0), and the aforementioned network tools. We generated Kaplan–Meier survival curves and time-dependent ROC curves with the R packages“survival” and “survival ROC,” respectively. Relevant immunological genes were identified using Spearman correlation analysis, and the threshold for statistical significance was set at *P* < .05.

## 3. Results

### 3.1. Association between DHRS1 and immune infiltration cells in HCC

**Figure 1.**
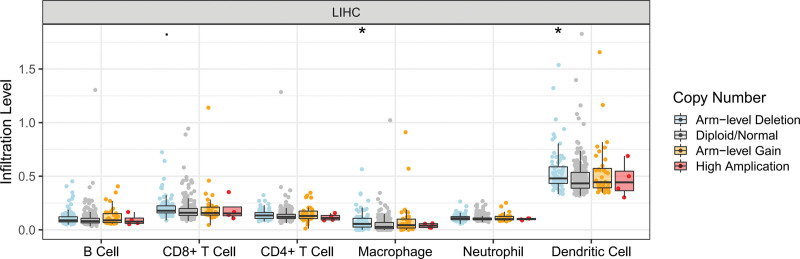
Immune relevance of DHRS1 in HCC based on tumor immune estimation resource (TIMER). Comparison of tumor infiltration levels among tumors with different DHRS1 copy number alterations*P < .05, **P < .01, ***P < .005. DHRS1 = a member of the short-chain dehydrogenase/reductase superfamily, HCC = hepatocellular carcinoma.

Infiltrated immune cells – key components of the TME – exert an impact on the prognosis of HCC patients. With limited evidence, Li et al reported that DHRS1 might contribute to the immune response in HCC.^[20]^ Here, we further investigated the relationship between DHRS1 and HCC immunity. In TIMER, several types of immune cell infiltration were found in LIHC, including macrophages and dendritic cells, and they appeared to be associated with changes in DHRS1 gene copy levels (Fig. 1). The expression of DHRS1 mRNA in LIHC was also positively correlated with some immune types and negatively correlated with others (Figure S1, Supplemental Digital Content, http://links.lww.com/MD/K397).

### 3.2. DHRS1 is associated with immune-related pathways in LIHC

**Figure 2.**
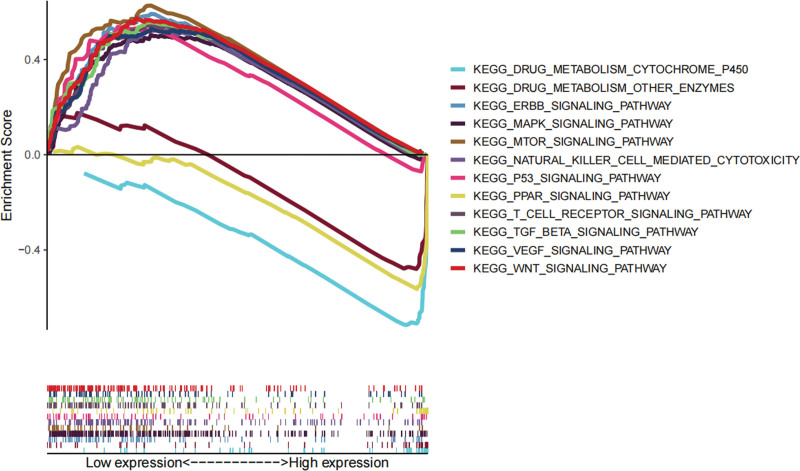
Representative signaling pathways for DHRS1 single gene GSEA analysis. DHRS1 = a member of the short-chain dehydrogenase/reductase superfamily, GSEA = gene set enrichment analysis.

In addition, we investigated potential signaling pathways regulated by DHRS1 in LIHC that may be responsible for modulating the immunological response. Several immune-related signaling pathways (including the MAPK signaling network, the mTOR-signaling pathway, and the P53-signaling system) were found to be associated with DHRS1 by gene set enrichment analysis (GSEA, Fig. 2), which validated that DHRS1 may affect tumor progression through immune-related pathways in HCC.

### 3.3. DHRS1 is associated with immunomodulators in HCC

**Figure 3.**
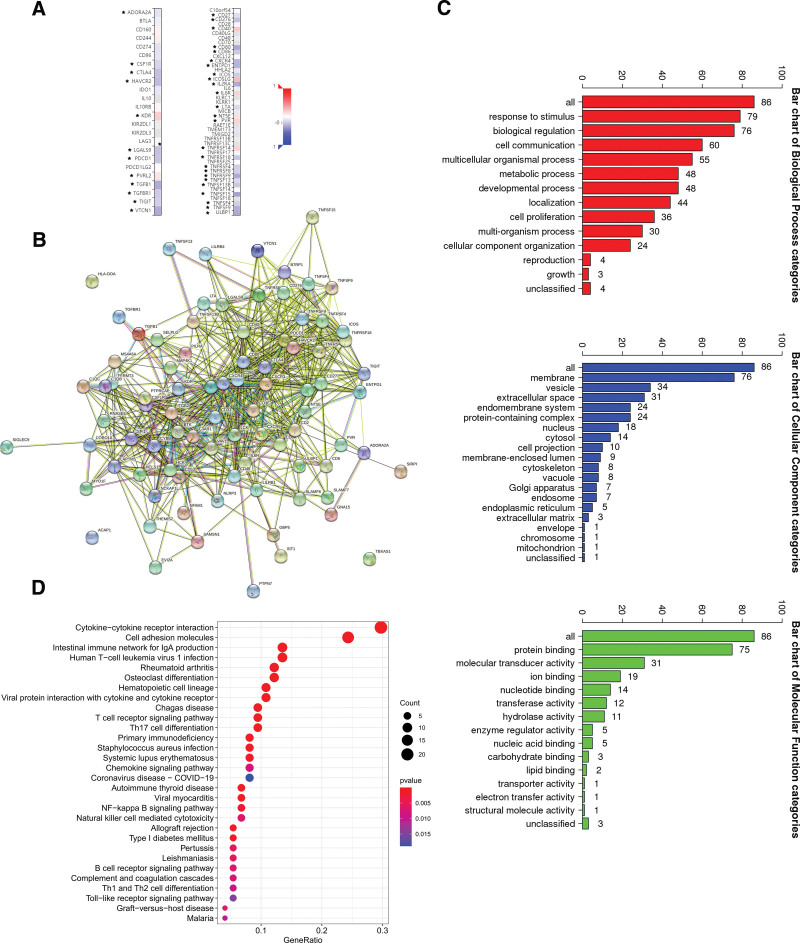
Identification and analysis of immunomodulators associated with the DHRS1 gene. (A) Immunostimulators (left panel) and immunoinhibitors (right panel) agents are significantly associated with DHRS1 in the LIHC. (B) Protein-protein network of 37 DHRS1-associated immunomodulators and 50 closely related genes in LIHC, produced by the STRING online server. (C) GO annotation of 37 DHRS1-associated immunomodulators and 50 closely connected genes in LIHC. (D) KEGG enrichment analysis of 37 DHRS1-associated immunomodulators and 50 closely related genes. *P < .05, **P < .01, ***P < .001. DHRS1 = a member of the short-chain dehydrogenase/reductase superfamily, STRING = the retrieval of interaction gene/proteins.

We next aimed to explore the relationship between DHRS1 and immunomodulators. We obtained 25 immunostimulators (CD27, CD276, CD40, CD80, CD86, CXCR4, ENTPD1, ICOS, ICOSLG, IL2RA, IL6R, LTA, PVR, NT5E, TNFRSF14, TNFRSF18, TNFRSF4, TNFRSF8, TNFRSF9, TNFSF13, TNFSF13B, TNFSF15, TNFSF4, TNFSF9, and ULBP1) and 12 immunoinhibitors (ADORA2A, CSF1R, CTLA4, HAVCR2, KDR, LGALS9, PDCD1, PVRL2, TGFB1, TGFBR1, TIGIT, and VTCN1) as agents that were significantly associated with DHRS1 in the LIHC (Fig. 3A). By constructing a protein-protein interaction network of the 37 immunomodulators, we obtain a close interaction relationship among DHRS1-related 50 immunoregulatory genes. (Fig. 3B). These genes were then annotated using GO terms, and subsequent KEGG analysis revealed that they played roles in immunological signaling pathways, such as T cell receptor signaling route and B cell receptor signaling pathway. (Fig. 3C–D)

### 3.4. Prognostic implications of DHRS1-associated immunomodulators in LIHC

**Figure 4.**
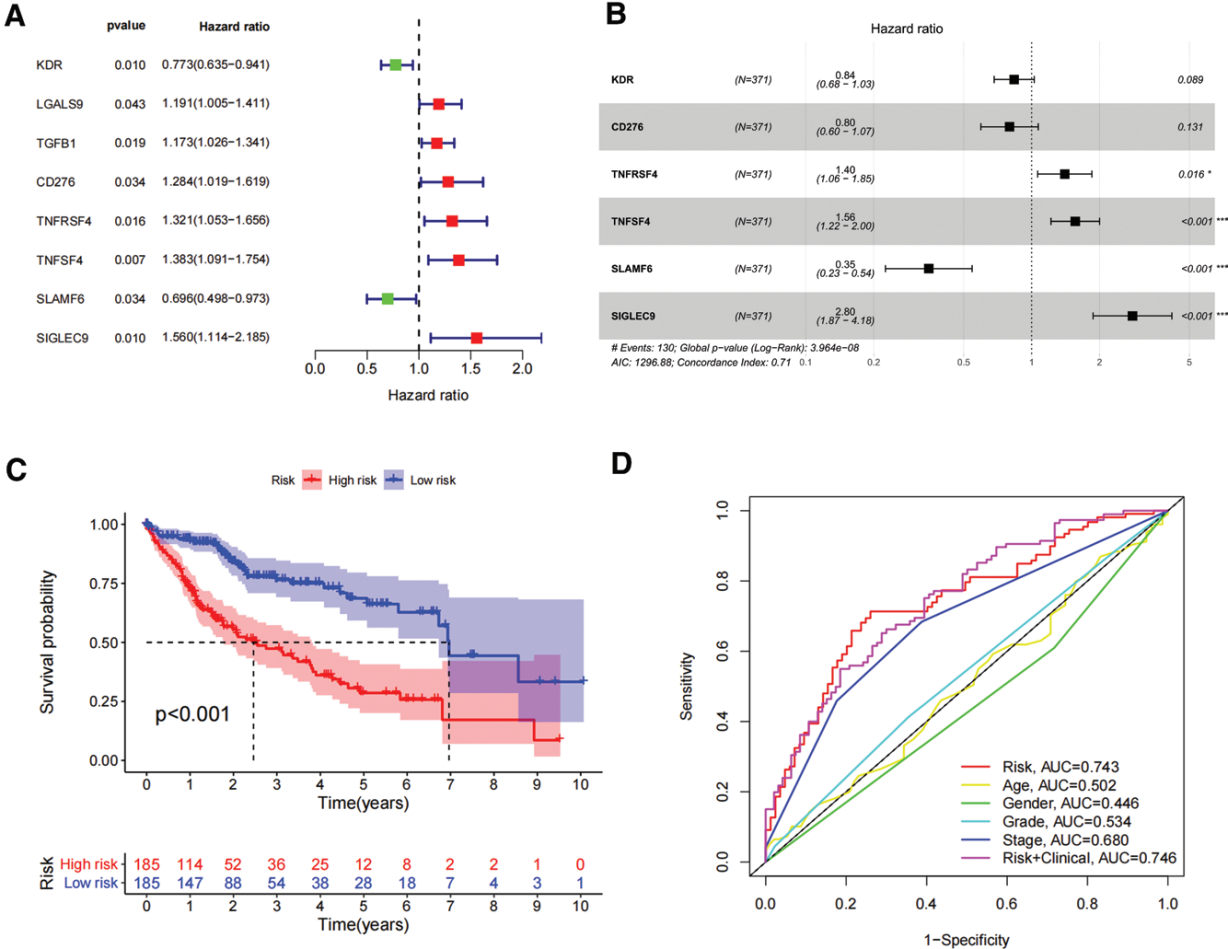
Develop prognostic gene signatures based on 87 DHRS1-related immune genes. (A) Univariate COX regression of DHRS1-associated immunomodulators. (B) Multivariate Cox regression analysis of DHRS1-associated immunomodulators. (C) Survival curves of the high and low risk groups of the TCGA set. (D) Time-dependent ROC curves of DHRS1-associated prognostic model of the TCGA set. DHRS1 = a member of the short-chain dehydrogenase/reductase superfamily, ROC = the receiver operating characteristic, TCGA = the cancer genome atlas.

**Figure 5.**
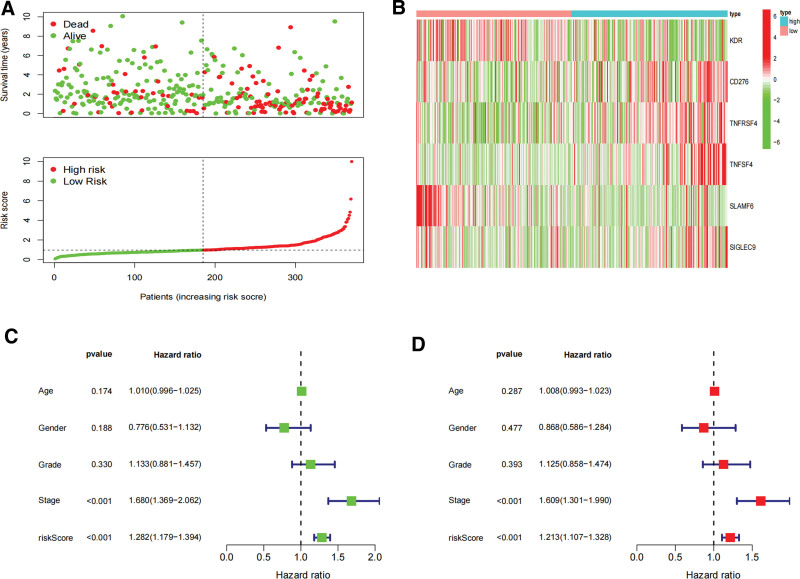
Characteristics of the DHRS1-associated prognostic signature and value in the TCGA dataset. (A) The distribution of risk scores and the survival status of HCC patients. (B) Heatmap of the DHRS1-associated prognostic signature expression profiles between the high and low risk groups. (C) Univariate Cox regression analyzes the relationship between risk score and overall survival. (D) Multivariate Cox regression analyzes the relationship between risk score and overall survival. *P < .05, **P < .01, ***P < .001. DHRS1 = a member of the short-chain dehydrogenase/reductase superfamily, HCC = hepatocellular carcinoma, TCGA = the cancer genome atlas.

**Figure 6.**
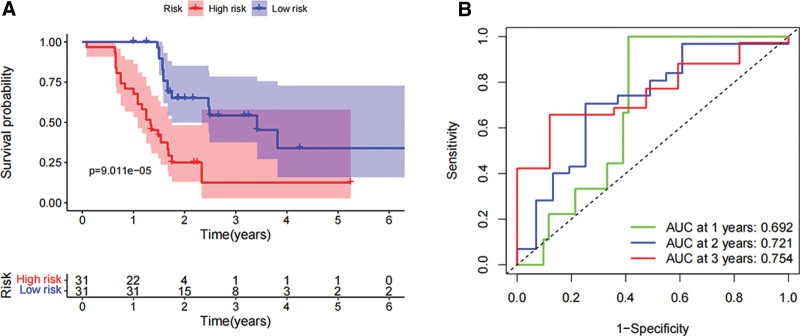
The identification of risk scores correlated with survival in patients with LIHC in the external validation set. (A) Survival analysis of the risk score in the external validation set. (B) ROC curves and area under the curve (AUC) for 1-, 2-, and 3-year survival in the external validation set. *P < .05, **P < .01, ***P < .001. ROC = the receiver operating characteristic.

In order to assess the prognostic value of 87 DHRS1-related immunomodulators, firstly, we selected 8 genes involved in prognosis using univariate Cox regression analysis (Fig. 4A), and then we identified 6 prognostic genes by multivariate Cox regression analysis (Fig. 4B). The expression coefficients of these 6 genes were as follows: KDR = −0.178, CD276 = −0.225, TNFRSF4 = 0.3388, TNFSF4 = 0.4457, SLAMF6 = −1.049, and SIGLEC9 = 1.0294. The risk scores were (−0.178 * X1) + (−0.225 * X2) + (0.3388 * X3) + (0.4457 * X4) + (−1.049 * X5) + (1.0294 * X6), with Xi representing each gene’s expression levels. The risk score was calculated by accumulating the product of these 6 genes expression and its coefficient in each sample. We downloaded the mRNA expression data of patients with TCGA-LIHC, and we noted that those patients with lower risk ratings fared much better than those with higher risk ratings using the Kaplan–Meier survival curve (log-rank test, *P* < .001) (Fig. 4C). The areas under the ROC curve (AUC) for risk score and staging were 0.743 and 0.680, respectively, and the AUC for the risk score and clinical score combined was 0.746 (Fig. 4D). The distribution of risk scores, survival status, and characteristic gene expression patterns of the HCC patients are illustrated in (Fig. 5A–B). Figure 5C displays the results of the univariate Cox regression analysis of the TCGA-LIHC, showing a statistically significant association between risk score and supplemental cont survival (hazard ratio = 1.282, 95% confidence interval = 1.179–1.394, *P* < .001). Our multivariate Cox regression analysis subsequently revealed that risk score was an independent predictor of HCC survival (hazard ratio = 1.213, 95% confidence interval of total = 1.107–1.328, *P* < .001) (Fig. 5D). Sixty-two HCC tissue samples were chosen for external validation to evaluate the validity of the risk score (see Table S2, Supplemental Digital Content, http://links.lww.com/MD/K399, which demonstrates the patient characteristics between high and low risk group). Moreover, the risk score of the 62 HCC patients at our center was calculated using the risk coefficients of the 6 genes in the experimental group, and the survival analysis was applied to verify the experimental group’s results. We thereby discovered that patients with lower risk scores possessed a significantly longer survival time and survival rate, consistent with the results from the training set (Fig. 6A). The AUCs for the biomarker-based prognostic model at 1, 2, and 3 years were 0.692, 0.721, and 0.754, respectively (Fig. 6B).

### 3.5. Construction of nomogram

**Figure 7.**
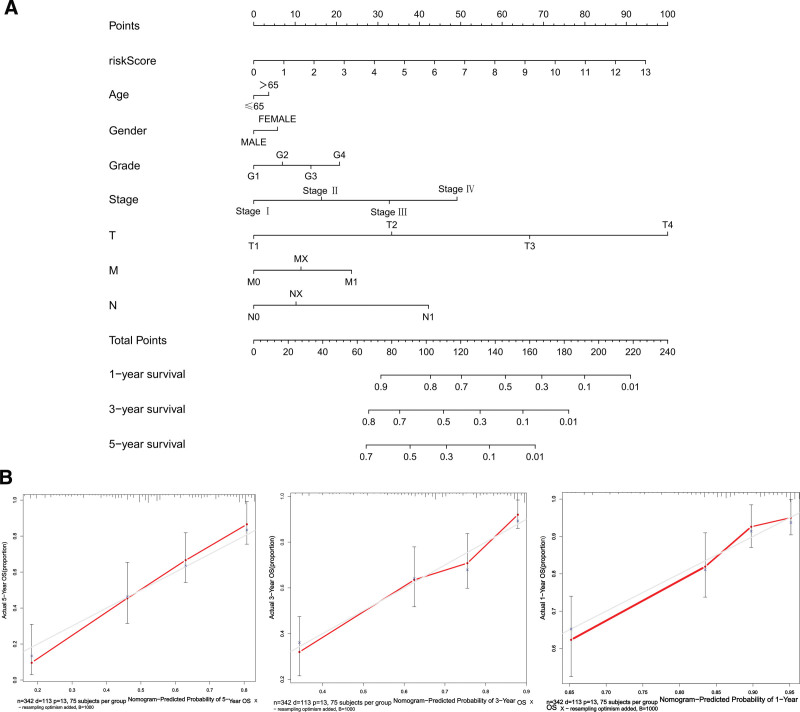
Establishment of the prognostic nomogram with the inclusion of the risk score. (A) A nomogram for predicting the overall survival of TCGA-LIHC patients. (B) Calibration curves of nomogram-predicted 1-, 3-, and 5-year survival of HCC patients. Red line: nomogram-predicted survival curve. Gray line: ideal survival reference curve. HCC = hepatocellular carcinoma, TCGA = the cancer genome atlas.

To estimate the probability of survival for HCC patients, we constructed a nomogram that allowed for risk score, stage, TNM, age, and sex (Fig. 7A). We calibrated the nomogram for 1-, 3-, and 5-year survival, and the calibration curve indicated a satisfactory match between the nomogram-predicted probability and the ideal reference line (Fig. 7B). Additionally, the concordance index was 0.71, which suggested good predictive power.

## 4. Discussion

In this study, we demonstrated that DHRS1 was closely associated with immunity in HCC, with DHRS1 expression linked to infiltrating immune cells and immunomodulators. We constructed multiple-gene risk-prediction signatures from the DHRS1-associated immunomodulators using stepwise COX regression and validated the prognostic model using clinical samples from our center. Finally, we used our prognostic model and clinical characteristics to generate an HCC prognostic nomogram for each patient that could then be used to guide HCC treatment after surgery.

Many members of the SDR family have been shown to display diverse functions in different illnesses; for example, DHRS12 has been used as an independent predictor of prognosis in cervical cancer patients.^[21]^ Furthermore, the activation of DHRS2 reduced the development of nasopharyngeal cancer by regulating lipid metabolism.^[22]^ By increasing Nrf2 ubiquitination, BDH2 (DHRS6) may augment endoplasmic reticulum stress and trigger cell death in gastric cancer.^[23]^ Liang et al^[24]^ ascertained that BDH2 reduced Bcl-2 expression, leading to elevated Bax levels and preventing HCC cells from proliferating. These findings collectively show that DHRS1 gene homologs are involved in cancer regulation.

Li et al^[25]^ reported low-level expression of DHRS1 in HCC tumor tissues and that DHRS1 mRNA and protein levels were associated with OS. DHRS1 levels drop as the tumor stage and grade rise, indicating the potential involvement of DHRS1 in HCC. However, how DHRS1 contributed to HCC was not previously understood. The TME is important in HCC development and therapeutic response, wherein cancer cells interact with multiple immune components to form a suppressive immune microenvironment that promotes immune escape, proliferation, invasion, and metastasis of HCC cells and that mediates drug resistance.^[26]^ We identified a correlation between DHRS1 gene copy number and infiltrating immune cells in pan-cancer via the TIMER database and demonstrated that the abundance of most immune subsets changed with the levels of DHRS1 mRNA using the TISIDB online tool. Evaluation of DHRS1-associated immunomodulators using TCGA-LIHC data with KEGG analysis suggests that the MAPK signaling pathway, the mTOR-signaling pathway, and the P53 signaling route are all involved in DHRS1-mediated immune responses.^[27]^ The roles of MAPK signaling in immunity and immunotherapy have been extensively studied. Activation of mTOR regulates tumor growth, metastasis, and immunity;^[28]^ and essential components of tumor immunology and homeostatic regulation of immune responses involve the p53 tumor suppressor pathway.^[29]^ Thus, the aforementioned results indicated a link between DHRS1 and immunity.

The liver is a powerful immune organ in the body, serving immune-defensive and immune-regulatory functions.^[30]^ TILs, including T cells and NK cells, are the immune cells that produce the greatest effects against tumors, and the impact of TILs on antitumor efficacy is proportional to their abundance and activity.^[31]^ TIL therapy was therefore developed to assess prognosis by directly identifying the targets of cancer cells. MITD1 gene expression was shown to be linked to immune infiltration in HCC, and studies by Shen et al^[32]^ have shown that it may serve as a predictive biomarker for Cox regression analysis. In the current study, we analyzed DHRS1 in a similar manner. We determined that multiple TILs and immunomodulators were abundantly associated with DHRS1, and using gene co-expression analysis, we observed potential mechanisms linking the high levels of TILs and immunomodulators to DHRS1. Multiple immunomodulator genes, such as KDR, TNFRSF4, CD276, TNFSF4, SLAMF6, and SIGLEC9, were shown to be co-expressed with DHRS1. Cox regression analysis revealed that KDR, CD276, and SLAMF6 were protective for HCC patients, while TNFRSF4, TNFSF4, and SIGLEC9 were risk factors.^[33]^ found SLAMF6 to be a predictive gene by adding the stromal and immune scores in HCC. Yoshiji et al^[34]^ depicted monotherapy with KDR/Flk-1 as boosting apoptosis in tumor cells – specifically in HCC cells. According to Cheng et al,^[35]^ patients with HCC who expressed the CD276 protein and developed vasculogenic mimicry reflected a poorer prognosis. Hong et al^[36]^ constructed an immune-related risk score model and uncovered a significant correlation between TNFSF4 and patient risk score. In a study of potential immunotherapeutic targets in HCC patients, Cai et al^[37]^ showed that the immune gene TNFRSF4 might comprise a promising prognostic biomarker for HCC. Ren et al^[38]^ also found that SIGLEC9 signaling was greatly reduced in HCC, suggesting that it might constitute an anti-oncogenic gene. Using DHRS1-related immunomodulators, we herein developed a predictive model that categorized HCC patients into high and low risk categories and produced a risk score that was substantially linked to survival.

To conduct external verification of risk score accuracy, we selected 62 HCC patients with tumor tissues for the present study. The mRNA expression levels of 6 immunomodulators were determined by qPCR, and these values were entered into the formula for the internal validation set. The survival curve was generated in combination with the clinical data, and the results of the external validation set were identical to the internal validation set. Furthermore, in the external validation set, we confirmed a correlation between DHRS1 expression and immunomodulators by qPCR. The above results suggest that the model established by DHRS1-related immunomodulators can be employed as an independent factor in predicting patient prognosis.

However, there were still limitations to the present study. First, this study was based on bioinformatics analysis of the TCGA-LIHC databases, and ascertaining whether DHRS1 actually regulates the immunomodulator functions requires further experimental research. Additionally, the number of samples used for external validation (62) was inadequate. Therefore, additional external studies are needed to confirm the results of our study.

## 5. Conclusion

DHRS1 may be involved in the regulatory process of the HCC immune microenvironment. Through analysis, we identified 6 prognostic immune genes associated with the immune microenvironment and HCC prognosis. The prognosis-predicted model generated by the 6 DHRS1-related immunomodulators could be adopted to anticipate the prognosis of HCC patients.

## Author contributions

**Conceptualization:** Yuxian Shen.

**Funding acquisition:** Hongchuan Zhao, Yuxian Shen.

**Methodology:** Wei Wang.

**Resources:** Sa Xu, Tao Meng, Fuyan Wang, Guoxing Wang, Fan Huang, Guobin Wang, Xiaojun Yu, Ruolin Wu, Liujin Hou, Xinghua Zhang.

**Software:** Sa Xu, Tao Meng, Fuyan Wang, Guoxing Wang, Fan Huang, Guobin Wang, Xiaojun Yu, Ruolin Wu, Liujin Hou, Zhenghui Ye, Xinghua Zhang, Wei Wang.

**Supervision:** Hongchuan Zhao, Yuxian Shen.

**Validation:** Hongchuan Zhao, Yuxian Shen.

**Visualization:** Sa Xu, Tao Meng, Fuyan Wang, Guoxing Wang, Fan Huang, Guobin Wang, Xiaojun Yu, Ruolin Wu, Liujin Hou, Zhenghui Ye, Xinghua Zhang.

**Writing – original draft:** Yuxian Shen.

**Writing – review & editing:** Guoxing Wang, Fan Huang, Guobin Wang, Xiaojun Yu, Ruolin Wu, Liujin Hou, Zhenghui Ye, Xinghua Zhang.

## Supplementary Material






