# Supplementary material for: Construction and validation of a immune-related prognostic gene DHRS1 in hepatocellular carcinoma based on bioinformatic analysis
Source: Medicine (Baltimore). 2023 Oct 20;102(42):e35268. doi: 10.1097/MD.0000000000035268 (PMC10589603; doi:10.1097/MD.0000000000035268)
Supplement: Supplementary file 1 [file medi-102-e35268-s001.docx]

Table 1 The primer sequences

| Gene | Human/mouse | Primer | Sequence (5’-3’) |
| --- | --- | --- | --- |
| **KDR** | Human | forward | 5'-GTGATCGGAAATGACACTGGAG-3' |
|  |  | reverse | 5'-CATGTTGGTCACTAACAGAAGCA-3' |
| **TNFRSF4** | Human | forward | 5'-GCAATAGCTCGGACGCAATCT-3', |
|  |  | reverse | 5'-GAGGGTCCCTGTGAGGTTCT-3' |
| **SLAMF6** | Human | forward | 5'-GAGTCCGCAAGGAACCTAGAG-3 |
|  |  | reverse | 5'-TCCCTGTTTGAATGAGTGACTGA-3' |
| **TNFSF4** | Human | forward | 5'-GGTCAGGTCTGTCAACTCCTT-3' |
|  |  | reverse | 5'-CATCCAGGGAGGTATTGTCAGT-3' |
| **SIGLEC9** | Human | forward | 5'-CCACATACCAAGAATTGCACCC-3' |
|  |  | reverse | 5'-ACAGAGAGCCGGTGATGTTTAT-3' |
| **CD276** | Human | forward | 5'-CTGGCTTTCGTGTGCTGGAGAA-3' |
|  |  | reverse | 5'-GCTGTCAGAGTGTTTCAGAGGC-3' |
| **GAPDH** | Human | forward | 5'-CCACTCCTCCACCTTTG-3' |
|  |  | reverse | 5'-CACCACCCTGTTGCTGT-3' |
