# Supplementary figures and images for: Construction and validation of a immune-related prognostic gene DHRS1 in hepatocellular carcinoma based on bioinformatic analysis
Source: Medicine (Baltimore). 2023 Oct 20;102(42):e35268. doi: 10.1097/MD.0000000000035268 (PMC10589603; doi:10.1097/MD.0000000000035268)

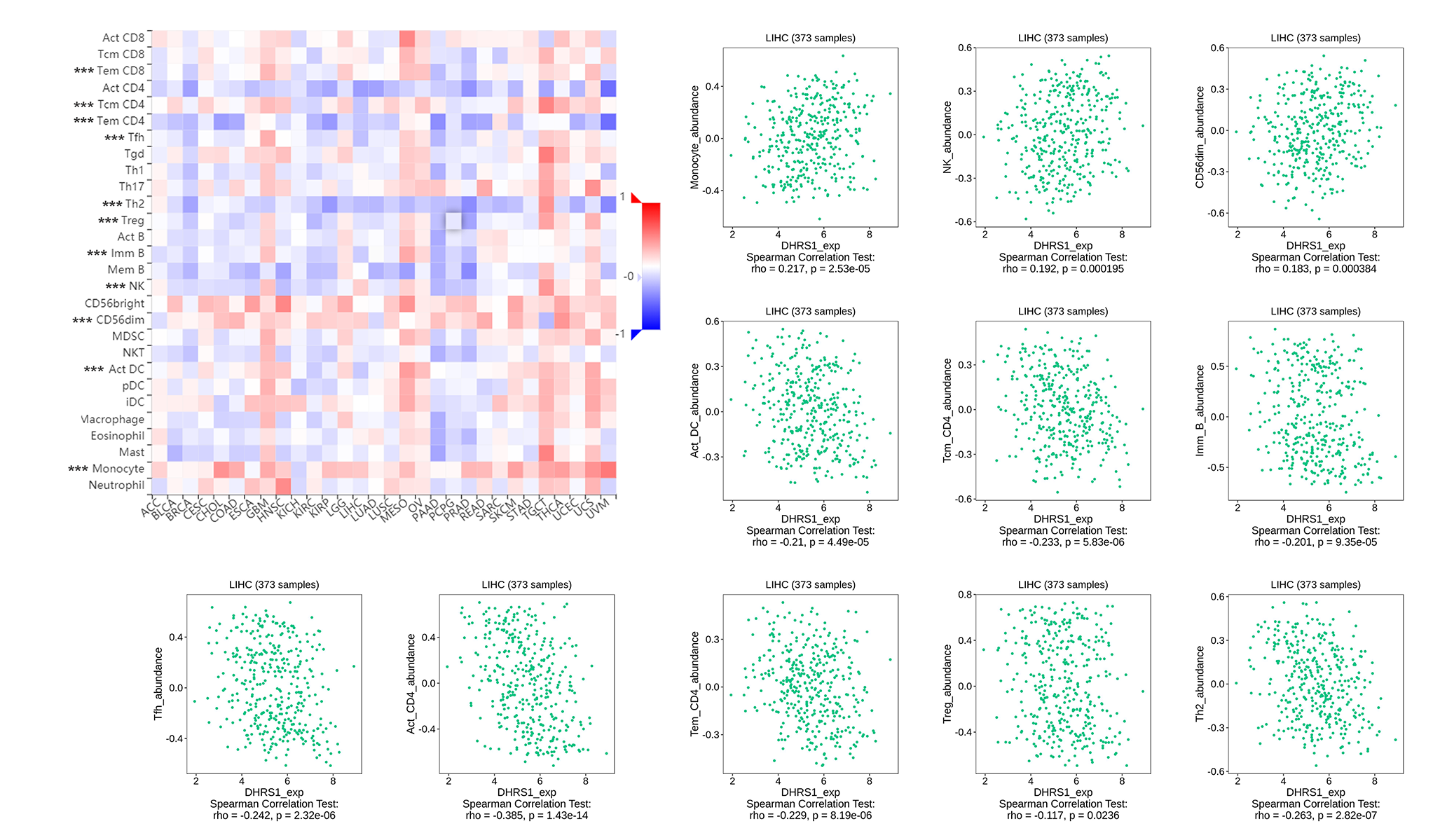

Supplement: Supplementary file 2 [file medi-102-e35268-s002.tif]
