# Supplementary material for: Construction and validation of a immune-related prognostic gene DHRS1 in hepatocellular carcinoma based on bioinformatic analysis
Source: Medicine (Baltimore). 2023 Oct 20;102(42):e35268. doi: 10.1097/MD.0000000000035268 (PMC10589603; doi:10.1097/MD.0000000000035268)
Supplement: Supplementary file 3 [file medi-102-e35268-s003.docx]

| Table 2 Patient Characteristics between high and low risk group | | | |
| --- | --- | --- | --- |
| Variable | High risk group (31) | Low risk group (31) | *P* value |
| Age | 54.0±12.7 | 61.1±10.6 | 0.019 |
| Tumor size | 9.6±4.7 | 5.6±2.5 | <0.01 |
| Gender |  |  |  |
| Male | 25（80.6） | 27（87.1） | 0.49 |
| Female | 6（19.4） | 4（12.9） |  |
| Smoking |  |  |  |
| Yes | 7 (24.1) | 9 (31.0) | 0.557 |
| No | 22 (75.9) | 20 (69.0) |  |
| Drinking |  |  |  |
| Yes | 7 (22.6) | 7 (22.6) | 1.0 |
| No | 24 (77.4) | 24 (77.4) |  |
| Cirrhosis |  |  |  |
| Yes | 17 (54.8) | 22 (71.0) | 0.189 |
| No | 14 (45.2) | 9 (29.0) |  |
| Multiple tumor |  |  |  |
| Yes | 4 (12.9) | 4 (12.9) | 1.0 |
| No | 27 (87.1) | 27 (87.1) |  |
| Vi |  |  |  |
| Yes | 4 (2.9) | 2 （6.7） | 0.414 |
| No | 27 (87.1) | 28 （93.3） |  |
| PVi |  |  |  |
| Yes | 9 (29.0) | 7 (22.6) | 0.562 |
| No | 22 (71.0) | 24 (77.4) |  |
| Lymphatic metastasis |  |  |  |
| Yes | 0 (0) | 1 (3.2) | 0.313 |
| No | 31 (100) | 30 (96.8) |  |
| Organonode metastasis |  |  |  |
| Yes | 0 (0) | 1 (3.2) | 0.313 |
| No | 31 (100) | 30 (96.8) |  |
| Distant metastasis |  |  |  |
| Yes | 1 (3.2) | 1 (3.2) | 1.0 |
| No | 30 (96.8) | 30 (96.8) |  |
| Stage |  |  |  |
| Ⅰ | 17 (54.8) | 20 (64.5) | 0.257 |
| Ⅱ | 1 (3.2) | 4 (12.9) |  |
| Ⅲ | 12 (38.7) | 6 (19.4) |  |
| Ⅳ | 1 (3.2) | 1 (3.2) |  |

*P < 0.05, **P < 0.01.
